# Traditional Chinese Medicine Decoction Combined With Antipsychotic for Chronic Schizophrenia Treatment: A Systematic Review and Meta-analysis

**DOI:** 10.3389/fphar.2020.616088

**Published:** 2021-01-20

**Authors:** Xiao-Jie Shi, Fang-Cheng Fan, Hua Liu, Yang-Wen Ai, Qing-Shan Liu, Yu-Guo Jiao, Yong Cheng

**Affiliations:** ^1^ Key Laboratory of Ethnomedicine of Ministry of Education, Center on Translational Neuroscience, School of Pharmacy, Minzu University of China, Beijing, China; ^2^ College of Life and Environmental Sciences, Minzu University of China, Beijing, China

**Keywords:** schizophrenia, TCM, antipsychotic, meta-analysis, adjuvant

## Abstract

Despite several studies suggesting the effectiveness of traditional Chinese medicine (TCM) in schizophrenia, there is still a lack of systematic summary and analysis on the role of TCM as adjuvant therapy in chronic schizophrenia. For this purpose, we conducted a meta-analysis to study the efficacy of TCM as an adjuvant combined with antipsychotics in the treatment of chronic schizophrenia. Until April 2020, based on the review of six electronic databases, eight articles were selected. The articles compared TCM decoction assisted antipsychotic therapies with an antipsychotic alone in the treatment of chronic schizophrenia by analyzing a total of 810 cases. The results showed that TCM combined with antipsychotics have beneficial effects on the Positive and Negative Syndrome Scale (PANSS), including the changes in total score, negative score, and the clinical effects evaluated by the PANSS scale. Subgroup analysis showed that the effects of auxiliary TCM with different efficacy on the positive and psychopathological scores were significantly different. It was found that adjuvant treatment with TCM can reduce some side effects and improve the patient's living conditions in the evaluation of the Schizophrenia Quality Of Life Scale (SQLS). Many studies have proved that TCM is safe and well-tolerated. Although the difficulties of using limited TCM remains to be generalized, it still has great potential in the adjuvant treatment of chronic schizophrenia.

## Introduction

Schizophrenia is a serious mental illness with a chronic course. It represents a large overall neurocognitive impairment (Steffen et al., 2020), characterized by positive symptoms (such as delusions), negative symptoms (such as emotional withdrawal), ideological and cognitive disorders (Waszkiewicz et al., 2012). The symptoms of schizophrenia are complex and diverse, with a range of pathophysiological mechanisms. Unfortunately, the exact cause of the disease is still unknown. People with schizophrenia have distinct subgroups, for example, drug-resistant diseases may be a distinct subtype of schizophrenia, and not just be a more severe form (Wimberley et al., 2016). The current antipsychotic regiment is effective in treating positive symptoms of the disease, but treatments to mitigate negative symptoms and cognitive areas are still limited (Mailman and Murthy, 2010; Maric et al., 2016). Clinicians usually encounter patients who show a series of symptoms. To improve the curative effect and solve all the symptoms, they usually add atypical antipsychotic drugs (other terms used to refer to these antipsychotics include “second generation” and “new antipsychotics”), which can improve negative symptoms, emotional symptoms and cognitive impairment in some cases (Bridler and Umbricht, 2003). Even in studies that have proven to be effective, a large number of patients are still unable to fully adapt to traditional treatments (Sant and Buckley, 2011). Most frequently, the problems in schizophrenia refer to social processing ability (i.e., social cognition) (Green et al., 2020). A small number of patients with schizophrenia recovered after the attack; however, the vast majority follow a chronic relapse process, unable to work for life (Meltzer, 1999). For chronic schizophrenia, although the efficacy and tolerance of antipsychotics have been covered before, people now focus on the overall clinical effectiveness of drug treatment in real life and expand upon the concept through functional recovery and self-evaluation of patients (Peuskens, 2004). Therefore, when the pathophysiology of different schizophrenia subtypes is not clarified, different treatment or adjuvant treatment strategies should be considered. We can continue to develop more effective interventions and make treatment more specific and targeted.

Traditional Chinese medicine (TCM) is a unique medical method gradually formed in long-term medical practice. Studies have shown that Chinese herbal medicine has been used to treat millions of people with schizophrenia for thousands of years. It may improve some prognosis of schizophrenia (Rathbone et al., 2007), and there is little evidence of side effects (Deng and Adams, 2016). Chinese herbal medicine can be used alone or in combination with other stimulants according to the patient's condition. The same drug and different dosage forms can have a great influence on the curative effect (Wang and Wei, 1995), so it allows flexible combinations. Some existing studies have shown TCM as an alternative therapy, or as a supplement to modern Western medicine, can effectively improve some outcomes of schizophrenia (Singh et al., 2010; Deng and Xu, 2016). To support the position and function of TCM in treating schizophrenia and assess the role of TCM as an auxiliary drug, this paper conducted system evaluation based on the randomized controlled trials (RCTs) of TCM decoction auxiliary treatment in chronic schizophrenia. Specifically, the purpose was to obtain evidence of the effect and safety of TCM in the adjuvant therapy of chronic schizophrenia and validating evidence-based medicine for its treatment.

## Methods

This systematic review and meta-analysis was performed according to the PRISMA (Preferred Reporting Items for Systematic reviews and Meta-analysis) guidelines (Moher et al., 2009). This study is a meta-analysis of the efficacy and safety of TCM in the treatment of chronic schizophrenia, excluding human studies.

### Database and Search Strategies

In this study, six databases were selected for all the randomized, controlled clinical trials concerning TCM decoction combined with antipsychotic drugs for the treatment of chronic schizophrenia. We conduct a systematic literature search in PubMed, Cochrane Library, EMBASE, Web of Science, China National Knowledge Infrastructure (CNKI), and Wanfang Data. The deadline for searching all the above electronic databases was April 3, 2020. To obtain more comprehensive literature, we searched the English database according to the following terms of “schizophrenia”, “schizophrenosis”, “Chinese medicine OR TCM OR combined with”, “randomized controlled trials OR RCT”. We used “精神分裂症”, “中药”, “联合 or 合并” to search in the Chinese electronic database. To reduce the risk of publication bias, search criteria were not limited by language date, document type, or publication status. By evaluating the title and abstract, we conducted a preliminary evaluation of the selected literature and passed a second evaluation of the full text. We finally extracted the studies concerning TCM decoction combined with antipsychotic drugs for the treatment of chronic schizophrenia.

### Inclusion and Exclusion Criteria

The inclusion criteria for this study are as follows: 1) Types of studies: All included studies are randomized controlled trials (RCT). 2) Diagnostic criteria: The patient was diagnosed with chronic schizophrenia according to ICD-10, CCMD-3, or Pathergasiology-5 (Shen, 2009), with an average duration of three years or more, no severe organic disease, and a total Positive and Negative Syndrome Scale (PANSS) score ≥60. There was no significant difference in general data between the control group and the experimental group. 3) Intervention type: Both the control group and the experimental group are treated with the same antipsychotic drugs. The type of TCM added in the experimental group was a decoction, which only played an auxiliary role, while the control group could add a placebo. 4) Outcome measures: The main outcome was the average change of overall symptoms of schizophrenia from baseline to endpoint through the Positive and Negative Symptoms Scale (PANSS). Other validated scales used to evaluate the overall symptoms of schizophrenia were the Schizophrenia Quality of Life Scale (SQLS) and Treatment Emergent Symptom Scale (TESS). The criteria “cure, significant effective, effective, or ineffective” was also included in the outcome measurement. Our exclusion criteria were as follows: 1) The admission diagnosis was unfounded or diagnosed with other types of schizophrenia. 2) The composition of adjuvant drugs was not clear (Only for self-made prescriptions). 3) The sample size of the study was small or the gender is single. 4) The grouping was not random.

### Data Extraction and Analysis

Two researchers (XJS, FCF) independently reviewed all the information provided by the electronic database, and all differences were resolved through negotiation. We have extracted the following information from the included studies: 1) The information of authors. 2) Year of publication. 3) Research location and method. 4) Study sample size. 5) Patient information. 6) Treatment intervention program. 7) Adjuvant effect. 8) Outcome measurements. 9) Outcome index.

### Quality Appraisal

The quality and bias risk of each included study were independently evaluated by two reviewers using Cochrane Collaboration's risk of the bias assessment tool. This tool can comprehensively evaluate the random method, blind method, and outcome index of the study. Any differences will be resolved in the discussion between the two researchers.

### Data Synthesis and Statistical Methods

Meta-analyses were performed using Review Manager 5.3 software for all calculations (Wallace et al., 2009). For continuous data, a weighted mean difference (WMD) with 95% confidence interval (CI) is calculated. According to the Heterogeneity of different trials, we chose a fixed-effect model or random effect model for further analysis. A *p* value < 0.05 was regarded as statistically significant. At least 50% of the evaluation of the heterogeneity of effect size through I^2^ statistic was considered as indicators of heterogeneity of results. Funnel plot analysis was used to detect publication bias.

## Results

### Study Selection

**FIGURE 1:**
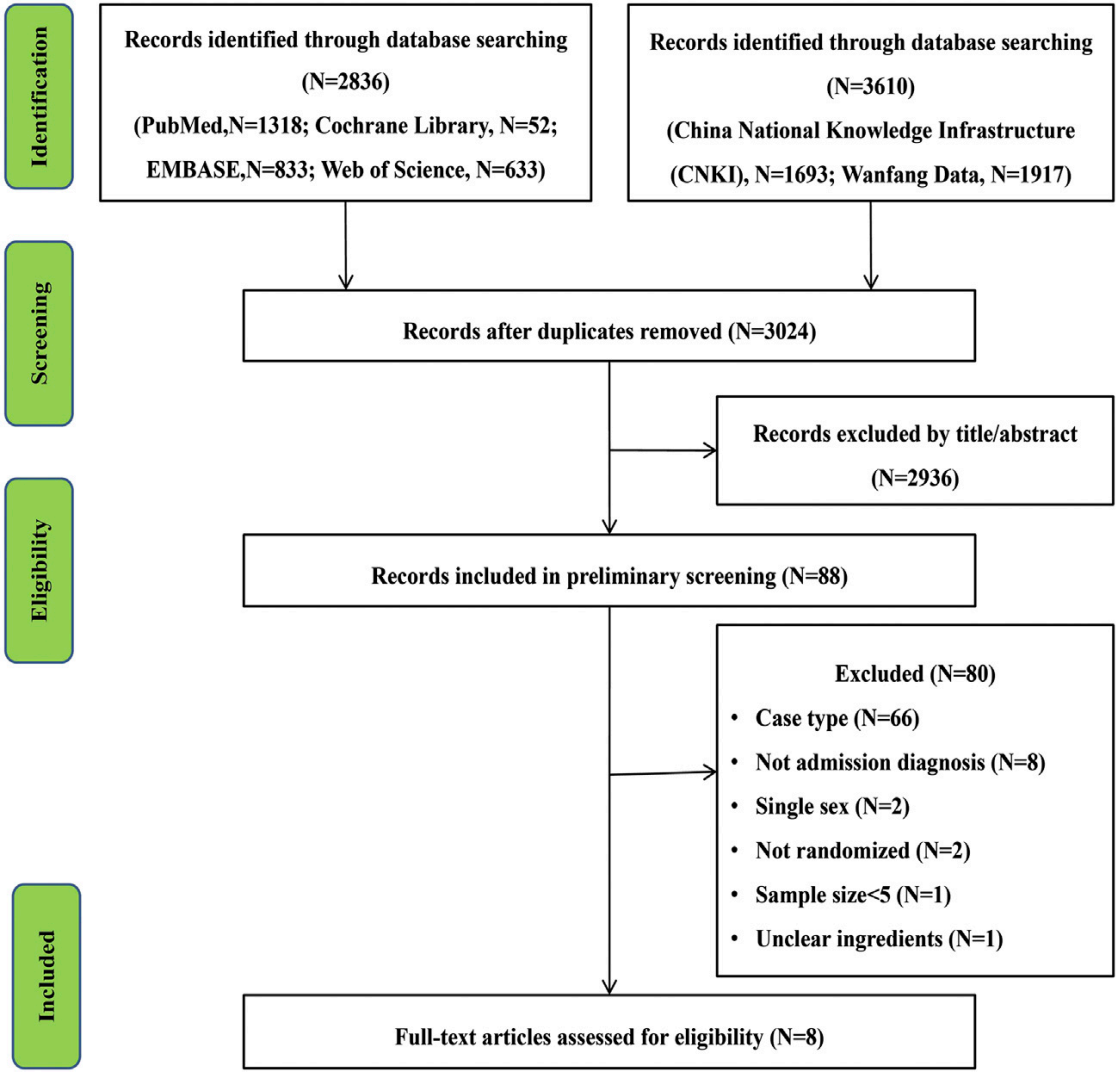
Flow chart of study selection.

After a preliminary screening of six electronic databases, 3,024 studies related to the research project were carried out after duplicate records were removed. After a further screening of titles and abstracts, 2,936 trials and 80 studies were excluded after an overall assessment of the full text. Finally, eight articles were included for our present meta-analysis (Liu et al., 2007; Zeng et al., 2007; Wang, 2008; Zhang, 2012; Han et al., 2014; Zeng et al., 2014; Zhang and Guo, 2017; Liu and Zhu, 2019). The screening process is shown in Figure 1.

### Characteristics of Included Studies

The total number of participants was 810 in the eight trials included (415 in the treatment group and 395 in the control group). There was no significant difference in clinical data between the two groups. The included studies were all single-center trials, but the treatment cycle was not consistent, as five studies were limited to 12 weeks (Liu et al., 2007; Zeng et al., 2007; Han et al., 2014; Zeng et al., 2014; Liu and Zhu, 2019) and three studies (Wang, 2008; Zhang, 2012; Zhang and Guo, 2017) were limited to 8 weeks. Detailed baseline information for inclusion in this study is listed in Table 1 and the information related to TCM decoction is shown in Supplementary Table S1 and Supplementary Table S2.

**TABLE 1:** Baseline information of the included studies.

Study	Location (country)	Diagnostic criteria	Method	Origin	Included (M/F)		Age (year) (M ± SD)		Duration of Illness (years)		Interventions (drug/dosage/frequency)		Adjuvant effect	Outcomes measurements
					T	C	T	C	T	C	T	C		
Zeng et al. (2007)	China	CCMD-3	12-weeks, double-blind, parallel-group	Inpatient	50 (22/28)	50 (24/26)	45.7 ± 15.6	45.4 ± 16.2	10.5 ± 3.5	9.9 ± 3.8	Aripiprazole/14.5 ± 5.5 mg/d; Jieyu Anshen decoction once daily	Aripiprazole/18.6 ± 6.8 mg/d; placebo formul once daily	A + B + C	PANSS/TESS
Liu et al. (2007)	China	CCMD-3	8-weeks, parallel-group	Inpatient	34 (21/13)	31 (15/16)	35.06 ± 8.16	36.97 ± 8.93	3.53 ± 2.16	3.81 ± 2.39	Risperidone/3.16 ± 0.73 mg/d; modified daotan decoction once daily	Risperidone/5.11 ± 1.27 mg/d	C	PANSS/TESS
Zhang and Guo (2017)	China	CCMD-3	12-weeks, parallel-group	Inpatient	59 (26/33)	58 (24/34)	45.39 ± 11.23	46.22 ± 10.19	9.83 ± 3.63	9.53 ± 3.82	Aripiprazole/10–25 mg/d; herbal decoction once daily	Aripiprazole/10–25 mg/d	A + B + C	PANSS/SQLS
Liu and Zhu (2019)	China	ICD-10	12-weeks, parallel-group	Inpatient	30 (17/13)	30 (15/15)	29.5 ± 4.0	30.0 ± 4.5	9.8 ± 1.3	10.2 ± 1.5	Aripiprazole/15–25 mg/d; Chinese medicine decoction once daily	Aripiprazole/15–25 mg/d	A + B + C	PANSS/SQLS
Wang (2008)	China	CCMD-3	8-weeks, parallel-group	Inpatient	86 (52/34)	74 (45/29)	36.26 ± 12.24	31.21 ± 16.52	3 – 46	4 – 48	Antipsychotic (N/A); Chinese medicine decoction once daily	Antipsychotic (N/A)	A + B + C	PANSS/TESS
Zhang (2012)	China	CCMD-3	8-weeks, parallel-group	Inpatient	36 (22/14)	32 (14/18)	36.06 ± 7.16	35.97 ± 9.82	3.63 ± 2.25	3.71 ± 2.49	Quetiapine Fumarate/0.2–0.6 g/d; modified daotan decoction once daily	Quetiapine Fumarate/0.6–1.0 g/d	C	PANSS/TESS
Zeng et al. (2014)	China	Pathergasiology-5	12-weeks, double-blind, parallel-group	Inpatient	60 (32/28)	60 (33/27)	46.75 ± 14.45	46.15 ± 14.85	11.55 ± 4.15	10.95 ± 3.85	Clozapine/505.15 ± 25.45 mg/d; Shu Gan decoction once daily	Clozapine/512.55 ± 35.85 mg/d; placebo formul once daily	A + B + C	PANSS/TESS
Han et al. (2014)	China	ICD-10	12-weeks, parallel-group	Inpatient	60 (27/33)	60 (26/34)	46.25 ± 15.86	46.19 ± 15.22	10.73 ± 3.64	10.55 ± 3.63	Aripiprazole/15–30 mg/d; Jieyu Anshen decoction once daily	Aripiprazole/15–30 mg/d	A + B + C	PANSS/TESS

CCMD-3, Chinese Mental Disorder Classification and Diagnosis, standard third edition; ICD-10, International Classification of Diseases-10th Revision; N/A, no detailed information; A, relieve qi; B, improve blood circulation; C, regulate water circulation; PANSS, positive and negative symptoms scale; TESS, treatment emergent symptoms scale; SQLS, the schizophrenia quality of life scale.

### Bias Risk of Included Studies

**FIGURE 2:**
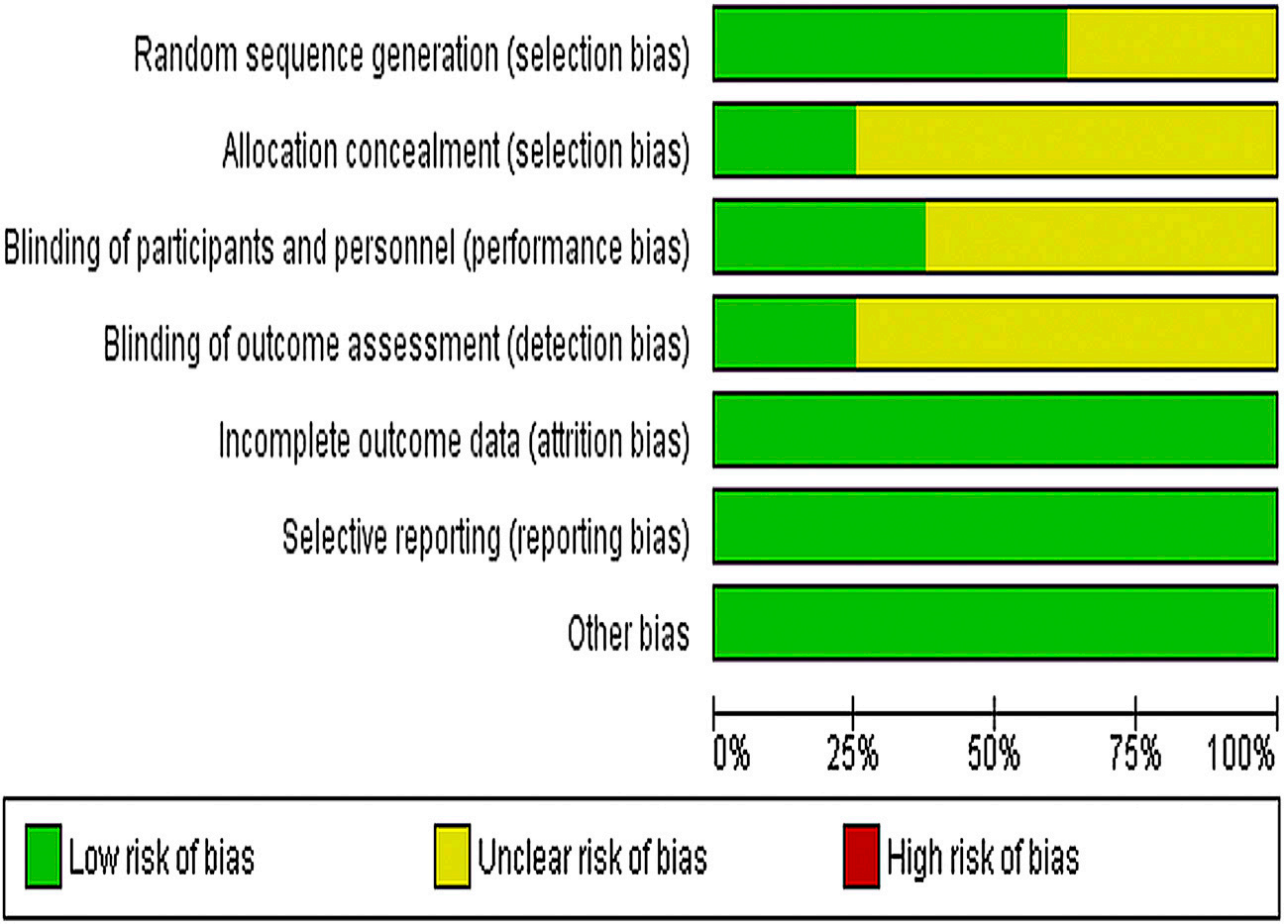
Risk of bias graph depicting each risk of bias item as percentages across all included studies.

We used Cochrane Collaboration's risk of the bias assessment tool to assess the risk of bias in the inclusion study, and the results are shown in Figure 2. All the eight experiments were randomly divided into groups, the stochastic methods include random number table sampling (Han et al., 2014; Zeng et al., 2007; Zeng et al., 2014) and random lottery (Liu et al., 2007; Zhang, 2012). Moreover, three studies were carried out without describing specific stochastic methods (Wang, 2008; Zhang and Guo, 2017; Liu and Zhu, 2019) and two studies were double-blind (Zeng et al., 2007; Zeng et al., 2014). All included studies were conducted in parallel, with participants being assessed before and after treatment.

### Synthesis of Results

All included trials evaluated the effectiveness of TCM plus antipsychotics vs antipsychotics alone. According to the different effects of TCM, two studies (Liu et al., 2007; Zhang, 2012) are “regulated water circulation”, while the others added “relieve qi” and “improve blood circulation”. All the included studies used PANSS scores to evaluate the efficacy.

#### Clinical Effects of TCM Plus Antipsychotics vs. Antipsychotics Alone

**FIGURE 3:**
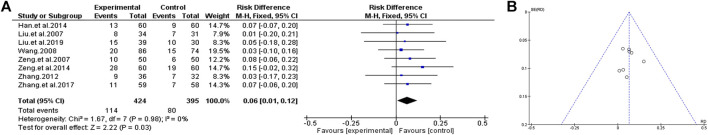
Clinical effects of TCM assisted antipsychotics vs antipsychotics therapy alone. **(A)** Forest plot. **(B)** Funnel plot.

We used the PANSS score to measure the clinical outcomes of TCM to assist antipsychotics vs. antipsychotics alone. Effects are divided into cured (≥75%), effective (25–70%), and ineffective (<25%) by reduction rate of PANSS total score before and after treatment. The cured patients were included in the curative group. We used the fixed-effect model to analyze the included studies, and the results showed homogeneity (χ^2^ = 1.67, *p* = 0.98, I^2^ = 0%). Compared to antipsychotics alone, treatment with TCM plus antipsychotics can significantly improve the clinical efficacy [MD = 0.06, (0.01, 0.12); Z = 2.22, *p* = 0.03], suggesting that TCM combined with antipsychotics therapy can help to improve the clinical efficacy of patients with chronic schizophrenia. As seen from the funnel plot, the research points are symmetrically distributed on both sides of the axis. The figure is symmetrical, and most of them are distributed at the top of the funnel. It is considered that the literature included in this study has no serious publication bias and is reliable (Figure 3).

#### PANSS Total Score Changes

**FIGURE 4:**
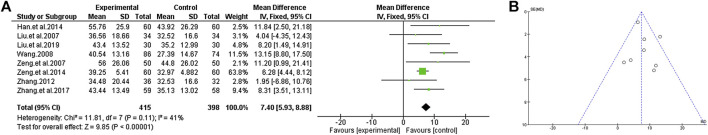
PANSS total score of TCM assisted antipsychotics vs antipsychotics therapy alone. **(A)** Forest plot. **(B)** Funnel plot.

We compared the efficacy of TCM plus antipsychotics with that of antipsychotics alone according to the changes of PANSS total score in the included trials. The analysis shows that there is a low significant heterogeneity in the consistency of the test results (χ^2^ = 11.81, *p* = 0.11; I^2^ = 41%), so the fixed effect model is used for statistical analysis to estimate the real value of the population. The analysis results showed that compared with the use of antipsychotics alone, TCM as an adjuvant therapy may have significant benefits in improving PANSS total scores [MD = 7.40 (5.93, 8.88); Z = 9.85, *p* < 0.00001] (Figure 4).

#### Negative Score

**FIGURE 5:**
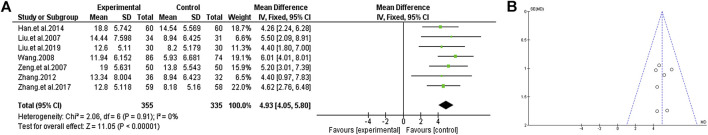
The negative score of TCM auxiliary antipsychotics vs. antipsychotics therapy alone. **(A)** Forest plot. **(B)** Funnel plot.

In total, seven studies reported negative symptoms with or without TCM auxiliary therapy. According to the heterogeneity score of the trial results, we select the fixed effects model for statistical analysis (χ^2^ = 2.06, *p* = 0.91; I^2^ = 0%). According to the analysis, the decrease of the negative score in the group of TCM plus the use of antipsychotics was greater than that in the group treated with antipsychotics alone, which indicated that the negative symptoms could be improved by increasing the adjuvant therapy of TCM [MD = 4.93 (4.05, 5.80); Z = 11.05, *p* < 0.00001] (Figure 5).

#### Positive Scale

**FIGURE 6:**
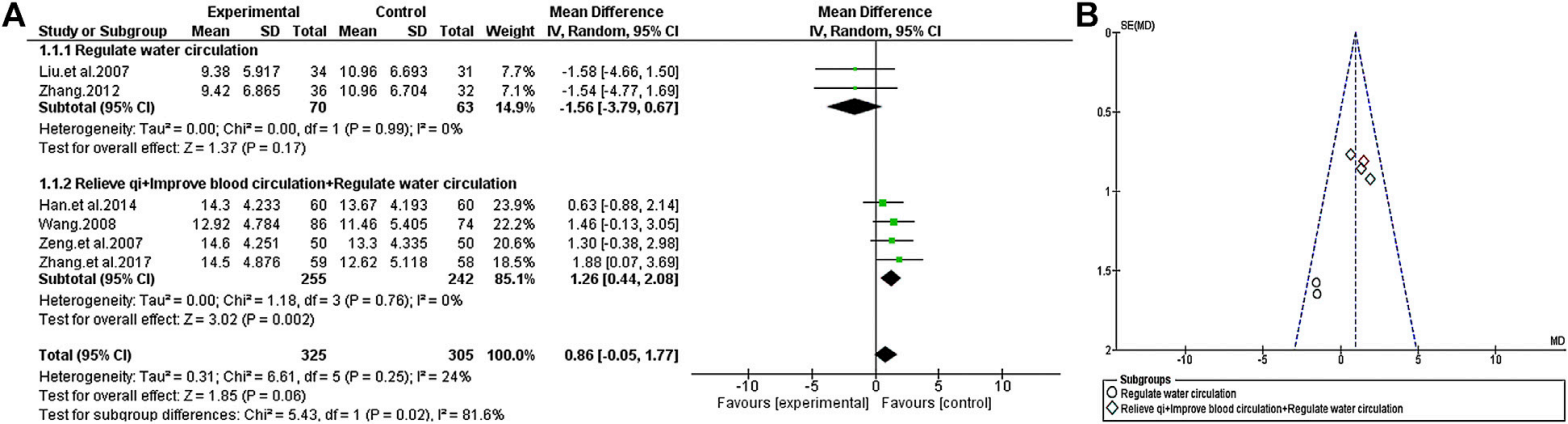
The positive scale of TCM assisted antipsychotics vs antipsychotics therapy alone. **(A)** Forest plot. **(B)** Funnel plot.

Except for Zeng et al., 2014, the curative effects of TCM plus antipsychotics and antipsychotics alone were compared by PANSS positive score in the other included trials. The consistency of the test results exhibited significant heterogeneity (χ^2^ = 31.60, *p* < 0.0001; I^2^ = 81%). Through further analysis, the heterogeneity of the consistency of the test results decreased after excluding the most extreme study (Liu and Zhu, 2019) (χ^2^ = 6.61, *p* = 0.25; I^2^ = 24%), so the random-effects model was selected for statistical analysis of the data. At the same time, to evaluate the causes of heterogeneity, we conducted a subgroup analysis of TCM according to its efficacy. Since all the tested auxiliary TCM included in the study have the effect of “regulate water circulation”, some of the TCM in studies also have the effect of “relieve qi” and “improve blood circulation”, so we conducted a subgroup analysis from this review. The results of subgroup analysis showed that compared with the group of antipsychotics alone, the use of auxiliary TCM in “regulate water circulation” could not improve the positive score [MD = −1.56 (−3.79, 0.67); Z = 1.37, *p* = 0.17], but increased the efficacy of “relieve qi” and “improve blood circulation” [MD = 1.26 (0.44, 2.08); Z = 3.02, *p* = 0.002] (Figure 6).

#### General Psychopathology Scale

**FIGURE 7:**
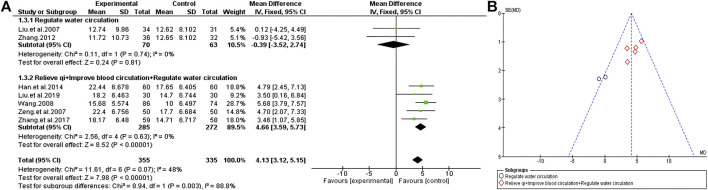
General Psychopathology scale of TCM assisted antipsychotics vs. antipsychotics therapy alone. **(A)** Forest plot. **(B)** Funnel plot.

A total of seven trials compared the effect of TCM plus antipsychotics therapy vs antipsychotics therapy alone through the changes in the general Psychopathology scale of PANSS. The results show that the consistency of the experimental results is relatively unified (χ^2^ = 11.61, *p* = 0.07; I^2^ = 48%), so the fixed effects model is selected for statistical analysis. Similarly, we conducted a subgroup analysis base on the efficacy of different TCM to find the source of heterogeneity. The results show that heterogeneity is related to the efficacy of adjuvant drugs. Compared with the simple antipsychotics treatment, the TCM adjuvant therapy with the function of “regulate water circulation” has no significant change on the general psychopathology scale [MD = −0.39 (−3.52, 2.74); Z = 0.24, *p* = 0.81], but the TCM adjuvant therapy with increased “relieve qi” and “improve blood circulation” effect has a very significant effect [MD = 4.66 (3.59, 5.73); Z = 8.52, *p* < 0.00001] (Figure 7).

#### Side Effects and Quality of Life

Although there are four trials with TESS scores (Table 2), the results show that there is no significant difference between TCM combine with antipsychotics therapy and antipsychotics alone, but the former therapy seems to ameliorate the score of the SQLS in some studies (Table 3). Among the two studies, the changes of scores in psychosocial, motivation and energy, symptoms and side-effects, and SQLS total score before and after treatment in the combined TCM group were significantly higher than those in the single antipsychotics treatment group.

**TABLE 2:** TESS scores after treatment.

Author (year)	Experimental			Control		
	Mean	SD	Total	Mean	SD	Total
Zeng et al. (2007)	2.4	1.1	50	2.2	1	50
Wang (2008)	3.95	2.36	86	3.97	2.29	74
Zeng et al. (2014)	12.08	3.01	60	12.18	3.24	60
Han et al. (2014)	2.38	1.03	60	2.26	1.01	60

**TABLE 3:** Analyses of SQLS score.

Study	Project	WMD	ConfIdence interval (95%)	*p* Value
Zhang and Guo (2017); Liu and Zhu (2019)	Psychosocial	3.10	[1.31, 4.89]	0.0007
	Motivation and energy	4.70	[1.81, 7.58]	0.001
	Symptoms and side-effects	3.99	[2.28, 5.70]	<0.00001
	Total	12.54	[7.25, 17.83]	<0.00001

## Discussion

In recent years, the role of TCM in global health care has been more understood by the scientific community. This scenario is evident through the increasing number of systematic reviews and meta-analysis that have been used to evaluate the efficacy of TCM to supplement and replace western medicine (Che et al., 2016; Wei et al., 2018). TCM treatment follows the concept of holistic practice, not only for a pathological phenomenon but to coordinate the physiological conditions of the human body to balance various indicators. Because of its unique overall treatment concept and personalized drug composition for different patients, it is a good adjuvant therapy which shows substantial promise to improve clinical outcomes. Western medicine mainly treats chronic schizophrenia with atypical antipsychotic drugs such as aripiprazole, risperidone, olazine, and typical antipsychotic drugs like chlorpromazine and haloperidol, which are effective for first-episode schizophrenia, but not ideal for chronic schizophrenia (Dimitrelis and Shankar, 2016). Numerous studies have suggested that the utilization of Chinese herbs in conjunction with western antipsychotics is beneficial in terms of mental state, holistic functioning, and decrease of adverse effects (Rathbone et al., 2005). Extant sudies show that TCM such as *Ginkgo biloba L*. and Wendan decoction (Singh et al., 2010; Deng and Adams, 2016) has beneficial effects on patients with schizophrenia. Thus, we found that TCM such as *Ginkgo biloba L.* extract (EGb), which enhanced the effectiveness of the antipsychotic drug haloperidol and reduced its extrapyramidal side effects (Zhang et al., 2001a), can also be used as an add-on therapy for chronic schizophrenia. EGb also has antioxidant properties and may improve the decreased peripheral immune function characteristic of schizophrenia (Zhang et al., 2001b; Zhang et al., 2006). Kim et al. (2018) found that *Panax ginseng* C.A. Meyer (PG) extract may be useful in neurodevelopmental disorder therapy, including psychiatric disorders such as schizophrenia. *Areca catechu* nuts, popularly known as “betel nuts”, possess antioxidant and anti-inflammatory effects and have beneficial effects on the positive, negative, and cognitive symptoms of schizophrenia (Adilijiang et al., 2015; Bales et al., 2009; Coppola and Mondola, 2012). *Areca catechu* nut extract could ameliorate memory impairment and cognitive decline by facilitating myelination processes in the frontal cortex (Adilijiang et al., 2015; Xu et al., 2019).

TCM is generally safe and well tolerated, but there is a lack of detailed research on the classification of diseases in Chinese herbal medicine forms. There are many dosage forms of Chinese herbal medicine, and are generally chosen according to the type of the disease and the nature of the drug and its components. Thus, for the same drug, the efficacy will be very different for different dosage forms. Because of the same drug appears in different forms of processing can have a great influence on the curative effect, we conducted a single dosage form and disease type as the research object, more targeted completion of the retrospective meta-analysis to look for evidence of TCM decoction as adjunctive therapy for chronic schizophrenia. Our results support the evidence that TCM decoction combined with antipsychotics can help improve the symptoms of chronic schizophrenia and, in some cases, improve the subjective quality of patients' life with schizophrenia. According to the analysis of existing research samples, TCM assisted antipsychotics can significantly improve the cure rate of chronic schizophrenia compared with the simple use of antipsychotics and has a significant effect on improving the total PANSS score and the negative score of patients. The heterogeneity in the analysis was solved by subgroup analysis according to the efficacy of auxiliary TCM. At the same time, we found that TCM with different efficacy played a different role in improving the PANSS score. Compared with patients who only received antipsychotic treatment, the addition of adjuvant TCM with efficacy “regulate water circulation” (such as Modified Daotan Decoction) could not improve the positive score and psychopathological condition, but adding “relieve qi” and “improve blood circulation” efficacy (such as Jieyu Anshen Decoction) to this function could significantly improve the above two conditions. This finding reminds us that due to the tendency of TCM to be individualized, efficacy drugs will be added according to the patient's situation, and the composition of TCM has higher freedom and pertinence. Combined with benefits, such as fewer side effects, it has great advantages as an auxiliary drug. It can be applied to the improvement of symptoms whose pathogenic mechanism is not clear. With the gradual formation of the bio-psycho-social medical model, quality of life is an important outcome measurement to judge disease impact and response to treatment (Guzmán et al., 1993). Through the analysis of the SQLS in two studies, it was found that patients treated with TCM could significantly improve their scores, but there was no difference between the four trials in the analysis of TESS, which may be limited by the number of studies.

Although our research results show that TCM assisted antipsychotic drugs in the treatment of schizophrenia have more positive effects in many aspects than antipsychotic drugs alone, due to some limitations, we should have a reservation about the overall safety and applicability of TCM adjuvant therapy. First of all, this analysis is affected by the limitations of the included study, because TCM therapy is popular in Asia, the condition of patients is affected by race. Therefore, it is necessary to evaluate its auxiliary effect objectively combined with the treatment situation in more regions. Secondly, the experimental methods included in the study still need to be more rigorous. Although all studies have adopted the method of random grouping, six studies did not pointed out a clear blind method that selection bias may increase since subjective factors. Finally, although TCM can be used as a useful adjuvant therapy and most studies have provided the drug composition and dosage of the decoction, there is no other detailed chemical analysis reported about specific herbs. However, the patients included in this study are all inpatients, and the quality of medicinal materials and the production of decoction are guaranteed. A subgroup analysis of complementary herbs was performed based on efficacy in this study, it did not limit the types of antipsychotics in the subgroup. These hidden drug information may be undescribed drug combination differences that influence potential treatment evaluation. A possible limitation is that even if the search follows a strict filter, eligible studies can be inadvertently missed. Therefore, more experimental research and a more perfect experimental design and detailed group analysis are needed to further support the positive role of TCM in the auxiliary treatment of chronic schizophrenia.

## Conclusion

Despite the differences in methodology and clinical samples among the included studies, the research design also has certain limitations. The existing analysis results still prove that the auxiliary treatment of chronic schizophrenia with TCM decoction has gratifying results. However, more evidence is needed to explore the improvement in psychiatric symptoms associated with different effects of Chinese herbal medicines to promote their wider use in combination with antipsychotics.

## Data Availability

The original contributions presented in the study are included in the article/Supplementary Material, further inquiries can be directed to the corresponding author.
