# Supplementary material for: Traditional Chinese Medicine Decoction Combined With Antipsychotic for Chronic Schizophrenia Treatment: A Systematic Review and Meta-analysis
Source: Front Pharmacol. 2021 Jan 20;11:616088. doi: 10.3389/fphar.2020.616088 (PMC7942273; doi:10.3389/fphar.2020.616088)
Supplement: Supplementary file 1 [file datasheet1.docx]

**Supplementary Table 1:** Composition of TCM decoction for included studies.

| **Study** | **Formulation** | **Source** | **Species, concentration** | **Quality control**  **reported?**  **(Y/N)** | **Chemical analysis**  **reported?**  **(Y/N)** |
| --- | --- | --- | --- | --- | --- |
| Zeng.et al.  (2007) | Jieyu Anshen  Decoction | The Second Hospital Affiliated to Xianning College | Root of *Bupleurum chinense DC.*, 10g  Root of *Curcuma aromatica Salisb.*, 10g  Root of *Conioselinum anthriscoides ‘Chuanxiong’* , 10g  Bark of *Paeonia × suffruticosa Andrews*, 10g  Root of *Pinellia ternata (Thunb.) Makino*, 10g  Root of *Arisaema heterophyllum Blume*, 10g  Root of *Smilax glabra Roxb.*, 15g  Peel of *Citrus × aurantium L.*, 10g  Root of *Paeonia lactiflora Pall.*, 10g  Root of *Rehmannia glutinosa (Gaertn.) DC.*, 10g  Seed of *Ziziphus jujuba Mill.*, 10g  Seed of *Platycladus orientalis (L.) Franco*, 12g  Root of *Polygala tenuifolia Willd.*, 10g  Root of *Aconitum kusnezoffii Rchb.*, 3g  Root of *Glycyrrhiza uralensis Fisch. ex DC.*, 10g  Stems of *Uncaria rhynchophylla (Miq.) Miq.*, 10g | Y- Prepared  according to Pharmacopoeia of the People's Republic of China | N |
| Liu.et al.  (2007) | Modified Daotan  Decoction | Kangning Hospital of Anshan City | Root of *Pinellia ternata (Thunb.) Makino*, 15g  Root of *Glycyrrhiza uralensis Fisch. ex DC.*, 10g  Peel of *Citrus × aurantium L.*, 15g  Root of *Bupleurum chinense DC.*, 15g  Root of *Curcuma aromatica Salisb.*, 15g  Root of *Arisaema heterophyllum Blume* with bile, 10g  Fructus of *Citrus × aurantium L.*, 15g  Root of *Smilax glabra Roxb.*, 15g  Root of *Codonopsis pilosula (Franch.) Nannf.*, 20g  Root of *Acorus calamus var. angustatus Besser*, 10g  Root of *Atractylodes macrocephala Koidz.*, 15g | Y- Prepared  according to Pharmacopoeia of the People's Republic of China | N |
| Zhang.et al.  (2017) | Herbal Decoction | Chenggu County Hospital of Traditional Chinese Medicine | Root of *Bupleurum chinense DC.*, 10g  Root of *Conioselinum anthriscoides ‘Chuanxiong’* , 10g  Seed of *Platycladus orientalis (L.) Franco*, 12g  Root of *Pinellia ternata (Thunb.) Makino*, 10g  Root of *Paeonia lactiflora Pall.*, 10g  Root of *Arisaema heterophyllum Blume* with bile, 10g  Seed of *Cyperus rotundus L.*, 10g  Root of *Smilax glabra Roxb.*, 15g  Root of *Glycyrrhiza uralensis Fisch. ex DC.*, 10g  Peel of *Citrus × aurantium L.*, 10g  Root of *Rehmannia glutinosa (Gaertn.) DC.*, 10g  Seed of *Ziziphus jujuba Mill.*, 10g  Root of *Polygala tenuifolia* *Willd*., 10g  Kernel of *Prunus persica (L.) Batsch*, 10g | Y- Prepared  according to Pharmacopoeia of the People's Republic of China | N |
| Liu.et al.  (2019) | Chinese medicine  Decoction | Department of Traditional Chinese Medicine Pharmacy, Yulin Mental Health Center | Root of *Conioselinum anthriscoides ‘Chuanxiong’ .*, 10g  Root of *Rehmannia glutinosa (Gaertn.) DC.*, 10g  Seed of *Ziziphus jujuba Mill.*, 10g  Seed of *Platycladus orientalis (L.) Franco*, 12g  Root of *Pinellia ternata (Thunb.) Makino*, 10g  Root of *Paeonia lactiflora Pall.*, 10g  Kernel of *Prunus persica (L.) Batsch*, 10g  Root of *Arisaema heterophyllum Blume* with bile., 10g  Seed of *Cyperus rotundus L.*, 10g  Root of *Glycyrrhiza uralensis Fisch. ex DC.*, 10g  Seed of *Citrus × aurantium L.*, 10g  Root of *Smilax glabra Roxb.*, 15g  Root of *Polygala tenuifolia Willd.*, 10g | Y- Prepared  according to Pharmacopoeia of the People's Republic of China | N |
| Wang.  (2008) | Chinese medicine  Decoction(Shunqi Daotan Decoction, Yangxin decoction, Xuefu Zhuyu Decoction) | The Sichuan Dazhou Kangfu Hospital | Not listed | Y- Prepared  according to Pharmacopoeia of the People's Republic of China | N |
| Zhang.  (2012) | Modified Daotan  Decoction | Shangqiu Second People' s Hospital | Root of *Pinellia ternata (Thunb.) Makino*, 15g  Root of *Arisaema heterophyllum Blume* with bile., 10g  Seed of *Citrus × aurantium L.*, 15g  Root of *Smilax glabra Roxb.*, 15g  Root of *Glycyrrhiza uralensis Fisch. ex DC.*, 10g  Fructus of *Citrus × aurantium L.*, 10g  Root of *Codonopsis pilosula (Franch.) Nannf.*, 20g  Root of *Atractylodes macrocephala Koidz.*, 15g  Root of *Acorus calamus var. angustatus Besser*, 15g  Root of *Bupleurum chinense DC.*, 15g  Root of *Curcuma aromatica Salisb.*, 15g  Seed of *Cyperus rotundus L.*, 15g  Drugs added as appropriate:  Seed of *Ziziphus jujuba Mill.*, 30g  Fructus of *Gardenia jasminoides J.Ellis*, 12g  Leaf of *Nelumbo nucifera Gaertn.*, 3g  *Cinnabar*, 0.3g  *Red bole*, 30g | Y- Prepared  according to Pharmacopoeia of the People's Republic of China | N |
| Zeng.et al.  (2014) | Shu Gan Decoction | Second Affiliated Hospital of Hubei University of Science and Technology | Root of *Bupleurum chinense DC.*  Root of *Curcuma aromatica Salisb*.  Root of *Conioselinum anthriscoides ‘Chuanxiong’*  Bark of *Paeonia × suffruticosa Andrews*  Root of *Pinellia ternata (Thunb.) Makino*  Root of *Arisaema heterophyllum Blume*  Root of *Smilax glabra Roxb.*  Peel of *Citrus × aurantium L.*  Root of *Paeonia lactiflora Pall.*  Root of *Rehmannia glutinosa (Gaertn.) DC.*  Seed of *Ziziphus jujuba Mill.*  Seed of *Platycladus orientalis (L.) Franco*  Root of *Polygala tenuifolia Willd*.  Root of *Aconitum kusnezoffii Rchb.*  Root of *Glycyrrhiza uralensis Fisch. ex DC.*  Stems of *Uncaria rhynchophylla (Miq.) Miq.* | Y- Prepared  according to Pharmacopoeia of the People's Republic of China | N |
| Han.et al.  (2014) | Jieyu Anshen  Decoction | Department of Psychiatric Shiling Hospital of Zhangjiakou | Root of *Bupleurum chinense DC.*, 9g  Root of *Curcuma aromatica Salisb*., 9g  Root of *Conioselinum anthriscoides ‘Chuanxiong’* , 9g  Bark of *Paeonia × suffruticosa Andrews*, 9g  Root of *Pinellia ternata (Thunb.) Makino*, 9g  Root of *Arisaema heterophyllum Blume*, 9g  Root of *Smilax glabra Roxb.*, 12g  Peel of *Citrus × aurantium L.*, 9g  Root of *Paeonia lactiflora Pall.*, 9g  Root of *Rehmannia glutinosa (Gaertn.) DC.*, 9g  Seed of *Ziziphus jujuba Mill.*, 9g  Seed of *Platycladus orientalis (L.) Franco*, 12g  Root of *Polygala tenuifolia Willd*., 9g  Root of *Aconitum kusnezoffii Rchb.*, 3g  Root of *Glycyrrhiza uralensis Fisch. ex DC.*, 9g  Stems of *Uncaria rhynchophylla (Miq.) Miq.*, 9g | Y- Prepared  according to Pharmacopoeia of the People's Republic of China | N |
