# Supplementary material for: Traditional Chinese Medicine Decoction Combined With Antipsychotic for Chronic Schizophrenia Treatment: A Systematic Review and Meta-analysis
Source: Front Pharmacol. 2021 Jan 20;11:616088. doi: 10.3389/fphar.2020.616088 (PMC7942273; doi:10.3389/fphar.2020.616088)
Supplement: Supplementary file 2 [file datasheet2.docx]

**Supplementary Table 2: Voucher specimens of TCM**

| **Scientific Name** | **Collection Time** | **Collection Location** | **Collector** | **Collector's No.** | **Save Location** |
| --- | --- | --- | --- | --- | --- |
| *Bupleurum chinense DC.* | 8/2/2005 | Shanxin Province. China | Li Qifa | 65 | Herbarium, College of life science , Henan normal university |
| *Curcuma aromatica Salisb.* | 11/12/2011 | Guangxi Zhuang autonomous region. China | Yang Jincai, Liao Yunbiao | LH1296 | Herbarium, Guangxi Institute of Botany,Chinese Academy of Sciences |
| *Conioselinum anthriscoides ‘Chuanxiong’* | 10/26/2014 | Hunan Province. China | Zhang Daigui | zdg4331270100 | Herbarium, Jishou University |
| *Paeonia × suffruticosa Andrews* | 4/30/1987 | Guizhou Province. China | Mao Fuben | 689 | Herbarium, Guizhou University of Traditional Chinese Medicine |
| *Pinellia ternata (Thunb.) Makino* | 7/22/2016 | Hunan Province. China | Zhang Daigui | 4331221605080361LY | Herbarium, Jishou University |
| *Arisaema heterophyllum Blume* | 8/12/2017 | Fujian Province. China | Hou Xueliang, Zhang Ruopeng | 1352 | Herbarium, School of Life Sciences, Xiamen University |
| *Smilax glabra Roxb.* | 24/5/2015 | Henan Province. China | Li Meijia | 21 | Herbarium, Henan Agricultural University |
| *Citrus × aurantium L.* | 7/28/1986 | Guizhou Province. China | Jia Anjing | 60 | Herbarium, Guizhou University of Traditional Chinese Medicine |
| *Paeonia lactiflora Pall.* | 6/29/2016 | Shanxi Province. China | Yang Hong | 1601042 | Herbarium, Shanxi University of Chinese Medicine |
| *Rehmannia glutinosa (Gaertn.) DC.* | 5/5/2017 | Hebei Province. China | Wang Mengzhen | 2017050501717 | Herbarium, Hebei Normal University |
| *Ziziphus jujuba Mill.* | 6/14/1959 | Shanxi Province. China | Guan Kejian | 5532 | Herbarium, Tianjin Natural History Museum |
| *Platycladus orientalis (L.) Franco* | 5/23/2016 | Guangdong Province. China | Dong Chuanhong | 20160012 | Herbarium, School of Life Sciences, South Chia Normal University |
| *Polygala tenuifolia Willd.* | 7/3/2016 | Shanxi Province. China | Wu Guan | 1624010 | Herbarium, Shanxi University of Chinese Medicine |
| *Aconitum kusnezoffii Rchb.* | 8/28/2008 | Henan Province. China | Wu Hao | A040 | Herbarium, Henan Agricultural University |
| *Glycyrrhiza uralensis Fisch. ex DC.* | 6/25/2016 | Inner Mongolia Autonomous Region. China | Zhang Chongling | 002 | Herbarium, Inner Mongolia Daxinganling forest investigation and Planning Institute |
| *Uncaria rhynchophylla (Miq.) Miq.* | 5/6/2015 | Hunan Province. China | Zhou Hui, Zhou Dasong | 15050632 | Herbarium, Forestry College, Central South University of Forestry and Technology |
| *Codonopsis pilosula (Franch.) Nannf.* | 6/29/2016 | Shanxi Province. China | Zhao Meng | 1610110 | Herbarium, Shanxi University of Chinese Medicine |
| *Acorus calamus var. angustatus Besser* | 5/22/2016 | Jiangsu Province. China | Hou Yuantong, Guo Chengyong, Hou Chunli | 201605555 | Herbarium, School of Life Sciences, Qufu Normal University |
| *Atractylodes macrocephala Koidz.* | 10/30/2015 | Anhui Province. China | Zhong Xin | CSH18552 | Shanghai Chen Shan Herbarium |
| *Cyperus rotundus L.* | 7/9/2017 | Guizhou Province. China | Pan Xiujuan | 201603021056 | Herbarium, Qiannan Normal University for Nationalities |
| *Prunus persica (L.) Batsch* | 3/28/2017 | Hebei Province. China | Zhang Wei | zw0085 | Herbarium, Hebei Normal University |
| *Gardenia jasminoides J.Ellis* | 7/9/2017 | Guizhou Province. China | Huang Mincong | 201603023059 | Herbarium, Qiannan Normal University for Nationalities |
| *Nelumbo nucifera Gaertn.* | 7/9/2013 | Jiangxi Province. China | Zhang Liping | 13306 | Jiujiang Forest Herbarium |
